# Functional antibodies against G-protein coupled receptors in Beagle dogs infected with two different strains of *Trypanosoma cruzi*

**DOI:** 10.3389/fimmu.2022.926682

**Published:** 2022-10-25

**Authors:** Gerd Wallukat, Fernando Antônio Botoni, Manoel Otávio da Costa Rocha, Vitória Louise, Johannes Müller, Andre Talvani

**Affiliations:** ^1^ Experimental and Clinical Research Center, Max-Delbrück Center for Molecular Medicine, Berlin, Germany; ^2^ Berlin Cures GmbH, Berlin, Germany; ^3^ Postgraduate Program in Infectiology and Tropical Medicine, School of Medicine, Federal University of Minas Gerais, Belo Horizonte, MG, Brazil; ^4^ Fundação Hospitalar do Estado de Minas Gerais, Belo Horizonte, MG, Brazil; ^5^ Department of Biological Sciences, Institute of Exact and Biological Sciences & Postgraduate Program of Health and Nutrition, School of Nutrition, Federal University of Ouro Preto, Ouro Preto, MG, Brazil

**Keywords:** *Trypanosoma cruzi*, dog model, chagas disease, autoantibodies, g-protein coupled receptors, Cardiomyocytes

## Abstract

The interaction of the anti-beta1-adrenergic receptor autoantibodies (β1ARAb) and the anti-muscarinic M2 receptor autoantibodies (M2RAb) with cardiac neurotransmitter receptors were identified in human chronic Chagas cardiomyopathy (CCC) related to the ECG and dysautonomia disturbances. Dogs are considered gold model to the study of *Trypanosoma cruzi* infection due the clinical similarities with CCC. This study aims to evaluate whether anti-β1ARAb, anti-β2ARAb, and anti-muscarinic M2RAb are generated in Beagle dogs infected by *T. cruzi* using Y and Berenice-78 strains of *T. cruzi.* Animals were infected with 4.0 x 10^3^ bloodstream trypomastigotes/kg of body weight and, after 25 months of infection, blood sample was collected, and serum stored at -80°C. Dog serum was treated by ammonium sulphate precipitation and the IgG antibodies isolated and added to the beating neonatal rats’ cardiomyocytes. All *T. cruzi*-infected dogs developed agonistic β1ARAb, β2ARAb, and M2RAb. Animals infected by Berenice strain presented less β2ARAb and M2RAb activities than dogs infected by Y strain of the parasite. In cardiomyocytes culture, the antibodies recognized an epitope on the second extracellular loop of the receptors which were similar to findings in human Chagas disease. There was no detection of antibody against G protein-coupled receptor in serum from uninfected dogs. In conclusion, both Y and Berenice-78 strains of *T. cruzi* induced dog antibodies, whose targets located in the second extracellular loop of the adrenergic and muscarinic receptors were similar to those observed in individuals with CCC. Therefore, our findings highlight dogs as a promisor model to investigate pathogenic roles of functional Ab against G-protein coupled receptors.

## Introduction

Chagas disease is caused by the protozoan *Trypanosoma cruzi* and is an important public health problem that still plagues 5.7 million people in Latin America and causes around 10.600 deaths annually (1). In Latin America, the prevalence of the disease has been decreasing and becoming more notable in Europe and the United States due to global immigration (2). Among clinical disturbances, chronic Chagas cardiomyopathy (CCC) is an important cause of heart failure and sudden death with a mortality rate of approximately 0.2-19.2%, depending on the population studied (3–5). The pathophysiology of CCC is still not completely understood and some hypotheses have been suggested (5). Myocyte destruction by the protozoan does not appear to be a dominant mechanism because parasite loads seem to be inadequate to induce the degree of observed tissue injury. Immune response against parasites and their antigens, and the autoreactivity against cardiac targets are evidenced in human and experimental models of *T. cruzi* infection (6–8), thus explaining the tissue damage. The autoimmune reactivity can be driven by molecular mimicry between host/parasite protein epitopes, which may occasionally exceed the threshold of acceptable immune self-tolerance, thus culminating in a cross-reaction against host cardiac molecules, and consequently promoting physiological disturbances and tissue destruction. In the beginning, a study conducted by Koberle suggested CCC as a neuronal cardiopathy primarily affecting the parasympathetic autonomous system (9), followed by others that described the cross-reactive host immune response against few *T. cruzi* antigens, homologous to cardiac and/or intestinal proteins (10–12).

The myocardial mononuclear infiltration and the almost absence of detectable parasites in tissue sections have supported the postulation of the autoimmune mechanisms playing a role in the development of CCC (8). Sterin-Borda et al. showed that anti-beta1-adrenergic receptor autoantibodies (anti-β1-AR Abs) were detected in the serum of patients with Chagas disease (10, 13). These anti-β1-AR Abs could stimulate cultured rat cardiomyocytes (14), and this stimulatory effect was inhibited by the β-blockade (13, 14). Anti-M2 muscarinic receptor autoantibodies (anti-M2R Ab) were later demonstrated in the serum of patients with CCC and found to diminish the contractile frequency of cultured mouse cardiomyocytes, an effect inhibited by a competitive inhibitor of acetylcholine, namely atropine (15). Wallukat et al. observed that the gamma globulin fraction in patients with idiopathic dilated cardiomyopathy (DCM) increased the beating rate of cultured rat cardiomyocytes, and this effect was blocked by propranolol (16). In contrast, Fu et al. demonstrated that the transfer of human lymphocytes from patients with DCM into SCID mice induced a disease similar to DCM (17). Later, Jahns et al. showed that isogenic injection of anti-β1ARAb in inbred rats induced non-inflammatory cardiomyopathy (18). These experimental data demonstrate that the agonistic activity of these autoantibodies against β1ARAb and M2RAbs may play an important role in the development of DCM.

Therefore, we assume the premises that (i) CCC pathophysiology is not yet completely understood, (ii) dogs develop myocarditis with arrhythmias, congestive heart failure, and dilated cardiomyopathy, similar to human clinical findings, and (iii) autoantibodies against G-protein coupled receptor (GPCR) contribute to the reduction of heart rate variability and cardiac dysautonomia (14, 19–23); thus, we aim to address whether these autoantibodies against GPCR are released under the presence of glycoproteins/antigens from genetically distinct *T. cruzi* populations in infected Beagle dogs.

## Material and methods

### Animals and *T. cruzi* infection

Sera obtained from Beagle dogs were kindly provided by Dr. Bahia from the Federal University of Ouro Preto (UFOP), MG, Brazil. Briefly, 4-month-old dewormed and vaccinated (Vanguard^®^ HTLP 5/CV-L - Pfizer) animals were intra-peritoneally infected with 4.0×10^3^ trypomastigote forms per kg of body weight of Berenice-78 (n=4) and Y (n=4) strains of *T. cruzi*. The infection was confirmed through fresh blood collected from the marginal ear vein of the dogs, and after 25 months of infection, 10 mL of blood was collected and serum was ultra-frozen at -80°C. Five age-matched non-infected dogs were used as controls. All procedures and experimental protocols were conducted in accordance with the procedures issued by the Brazilian College of Animal Experimentation (COBEA) and approved by the Ethics Committee in Animal Research at UFOP as informed by Guedes et al. (23).

### Neonatal rat ventricular myocyte culture

The neonatal rat cardiomyocytes were prepared from the cardiac ventricle from 3 days old Wistar rats as described by Wallukat et al. (24). All procedures involving rats and cardiomyocytes were conducted in accordance with ethical procedures approved by the Ethics Committee in Research (# Y9008/12 and # Tötungsanzeige Y9004/19), at the Max Delbrück Centre for Molecular Medicine Berlin, Germany. Briefly, the ventricles were dissected into 1 mm^2^-sized fragments with two scalpels in a Ca^++^-free phosphate-buffered saline (PBS) solution. After washing, the ventricular fragments were transferred to 10 mL of Ca^++^-free PBS containing 0.2% crude trypsin in an Erlenmeyer flask. The heart fragments were slowly stirred on the desk of a magnetic stirrer for 15 min at 37°C. The supernatant was added to a plastic conical tube [BD, Falcon] (50 mL) containing 5 mL of ice-cold neonatal calf serum. The tube was centrifuged at 130xg for 15 min. Next, the supernatant was discarded, and the pellet was dissolved in a complete SM 20-I cell culture medium. This procedure was repeated three times, following which the cell containing SM 20-I samples were collected and centrifuged again. The pellet was dissolved into a complete SM 20-I culture medium containing 0.5 µM fluorodeoxyuridine to prevent the over-growth of non-myocytes (25). The cells were seeded in cell culture flasks [BD, Falcon] (12.5 cm^2^) at a density of 2.4×10^6^ cells/2 mL. The culture medium was replaced after 24 h and then, on every second day. The cardiomyocytes started spontaneously beating after 2 days of culture. The beating cardiomyocytes were cultured for up to 10 days at 37°C.

### *In vitro* rat cardiomyocytes stimulation

The beating rate of the spontaneously beating cardiomyocytes was measured on a heated desk (37°C) using an IonOptix system (IonOptix LLC, Westwood, MA, USA) and a Carl Zeiss Axio Observer A1 microscope (Carl Zeiss AG, Oberkochen, Germany). First, the basal beating rate of the cardiomyocytes was based on low-impedance microelectrode devices, arranged in a grid, and designed for noninvasive recordings at six sites across the culture flask. Second, the drugs or Abs were added to the culture medium and the beating rate was measured again on the corresponding mark after 5 or 60 min, respectively. Cardiomyocytes with a basal beating rate between 100 and 200 beats/min were used in the experiments (26). The receptor specificity of the Abs was determined by using specific receptor antagonists or peptides corresponding to the extracellular loops of the GPCR. The Abs identified in individuals with Chagas disease are directed against the second extracellular loop of these receptors. Therefore, we used 5 to 6 short overlapping peptides corresponding to extracellular structures of the human β1-AR, β2-AR, or M2R for the identification of the epitopes localized into the second extracellular loop. The second extracellular loop of the three investigated receptors exhibits a high homology among dogs, rats, and humans. The peptides that neutralize the agonistic activity of the Abs represent the epitope in this loop that react with the antibody.

### Immunoglobulin preparation

The isotype of the immunoglobulin (IgG) was isolated from the dog sera as previously described for the human IgG (25). The dog sera (0.5 mL) were treated with 0.33 mL saturated ammonium sulfate, mixed, and incubated overnight at 4°C. Next, the samples were centrifuged at 2795xg. The supernatant was discarded, and the pellet was dissolved in 0.5 mL physiologic NaCl solution buffered with phosphate buffer (pH 7.4). Following that, 0.5 mL ammonium sulfate was added to precipitate the Igs again and the samples were centrifuged again. This step of precipitation and centrifugation was repeated once. The resulting pellet was dissolved in 0.5 mL of the buffer and extensively dialyzed against 1 L of this buffer at 4°C. The buffer was refreshed four times in 4 days. The IgGs were stored at -20°C. All Ig preparations were previously evaluated in a dose-response curve for affinity, and the ideal pharmacological dilutions (1:40 or 1:50) were identified for the crude antibody in this study (27).

### Statistical analysis

All data in this study were presented as mean ± standard error of the mean. Owing to our small sample size, we performed a pairwise non-parametric Kruskal–Wallis test to examine the statistical significance.

## Results

**Figure 1:**
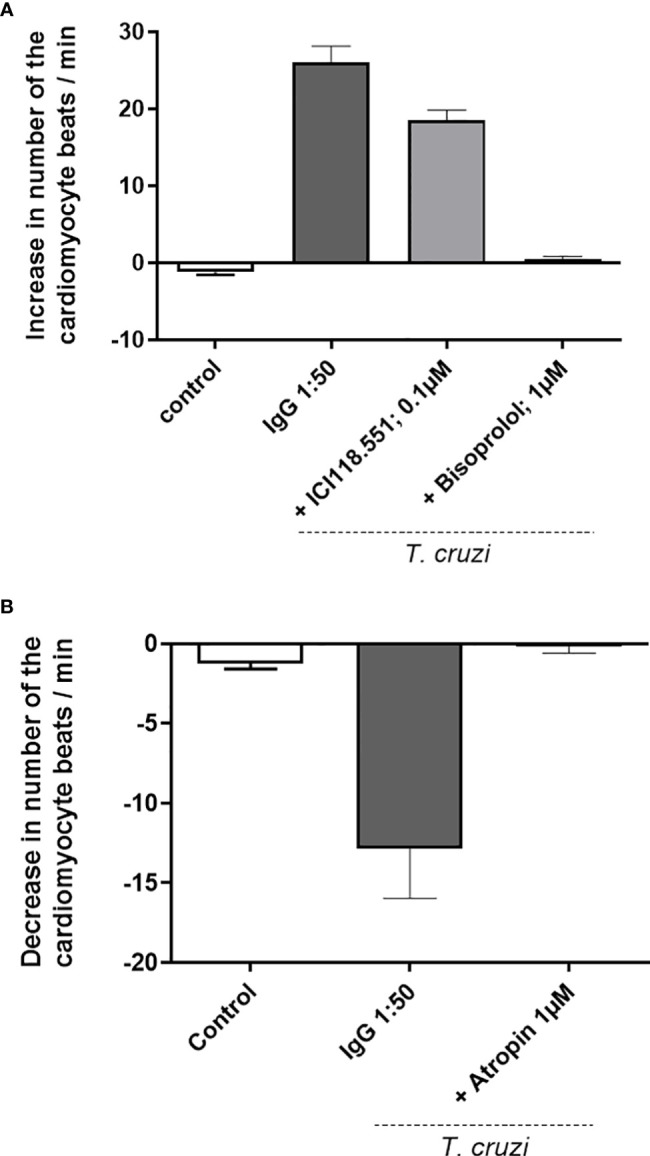
**(A)** Positive chronotropic response of the cardiomyocytes after the addition of the immunoglobulin G (IgG) preparation, obtained from dogs infected with the Y-strain, in the presence of atropine. The cardiomyocytes show an accelerated beating frequency, and the effect of IgG was blocked by specific antagonists of the β2-adrenergic receptor (AR) (ICI118.551) and antagonist of the β1-AR (bisoprolol). The IgG of uninfected control animals exert no effect. **(B)** IgG preparation from dogs infected by Y-strain under blockade of the β1-AR and β2-AR by bisoprolol and ICI118.551, respectively. Under these conditions, the cardiomyocytes response with a negative chronotropic effect induced *via* the muscarinic M2 receptor and blocked by atropine.

**Figure 2:**
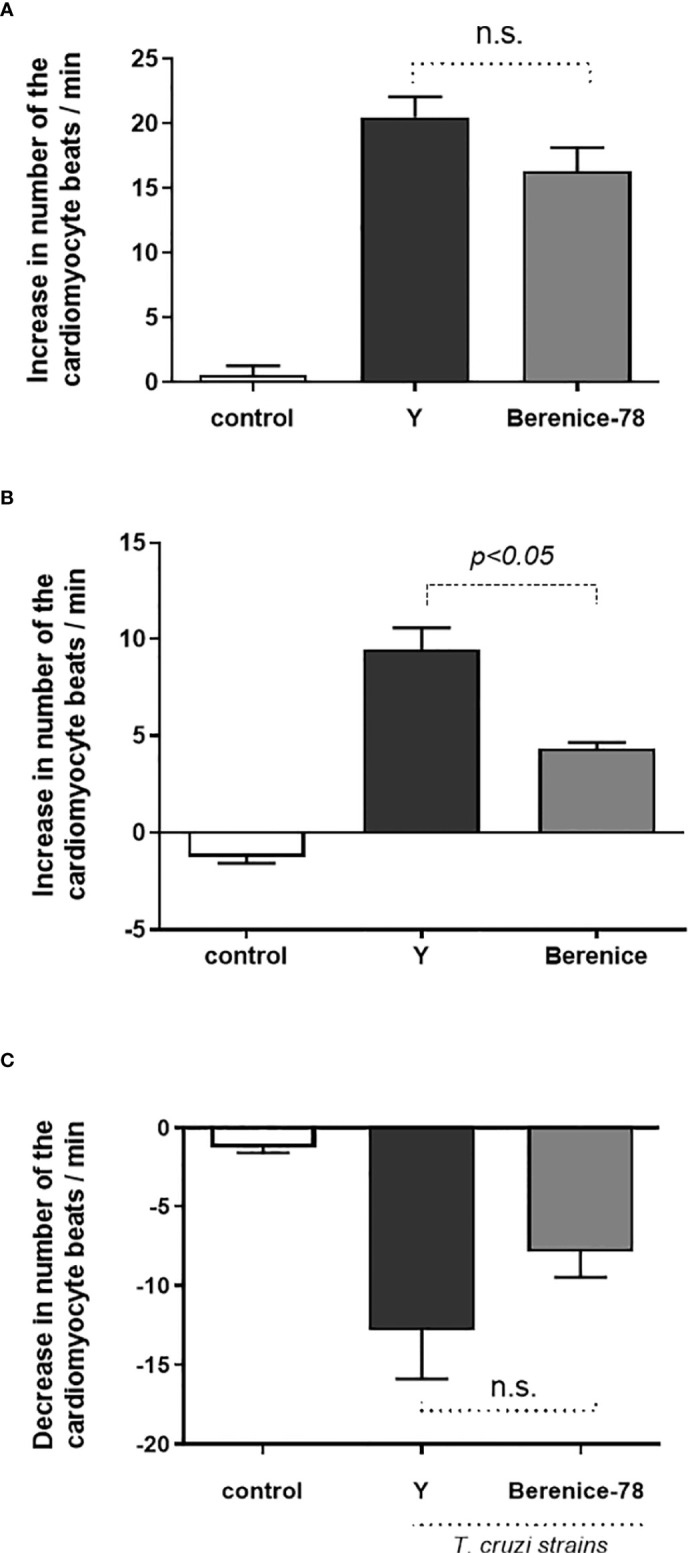
Activity of the β1-adrenoceptor, β2-adrenoceptor and muscarinic M2 receptor antibodies (Abs) on serum obtained from animals infected by Y and Berenice-78 *T. cruzi* strains and on serum from uninfected dogs (controls). In the control samples, no functional Abs against G-protein coupled receptor were identified. **(A)** β1-adrenoceptor Abs activity; **(B)** β2-adrenoceptor Abs activity; and **(C)** the negative chronotropic activity of the muscarinic M2 receptor Abs. N.S., not significant (p>0.05).

Our data demonstrate that dogs infected with Y and Berenice-78 strains of *T. cruzi* developed functional autoantibodies against GPCRs. These functional Abs are directed against β1-AR, β2-AR, and M2R. **Figure 1A** shows the effects induced by the Igs obtained from dogs infected with the Y-strain added at a dilution of 1:50 in the presence of atropine. The Igs exert a positive chronotropic effect that was partially blocked by the β2-AR antagonist ICI-118.551 and completely blocked by the β1-AR antagonist bisoprolol. In contrast, the Igs induced a negative chronotropic effect when the cells were pretreated with the antagonists of the β1-AR and β2-AR. This effect was blocked by atropine, an antagonist of the M2R. (**Figure 1B**). These data indicate that dogs infected with both strains of *T. cruzi* developed functional Abs against GPCR. No functional Abs were identified in the uninfected control dogs presenting the same biological age. It seems that animals infected with the Berenice-78 strain developed lesser Ab activity against GPCR than those dogs infected with the Y-strain (**Figures 2A–C**). The β2-AR Ab activity of animals treated with Ab obtained from dogs infected with the Y-strain was higher than that of animals treated with Ab obtained from dogs infected with the Berenice-78 strain (p < 0.05, **Figure 2B**).

Our data indicate that Abs obtained from dogs infected with Y-strain of *T. cruzi* presented 69% of functional activity *via* β1-AR and 31% *via* β2-AR. In contrast, parasites of the Berenice-78 strain induced a lower Abs activity, induced by the adrenergic response, with 79% *via* β1-AR and 21% *via* β2-AR. The β2-AR Ab activity in the Y strain-infected animals was significantly higher than that in the Berenice-78 strain-infected animals. The change in cardiomyocyte beating rate was 9.2 Δ beats/min related to the Y-Strain and 4.6 Δ beats/min related to the Berenice-78 strain (p < 0.05). For the negative chronotropic effect induced *via* Abs, we determined a change of -12.8 Δ beats/min and -7.8 Δ beats/min related to the Y and Berenice-78 strains, respectively.

We further showed that under stimulation by both strains of the parasite, the functional Abs against adrenergic receptors were equally synthesized. However, the effects triggered by the Y-strain *via* the β1-AR seemed to be higher than those triggered by the Berenice-78 strain of *T. cruzi*. A similar result was observed in the effects *via* the β2-AR, where the effects were 31% higher related to the Y-strain than those related to the Berenice-78 strain (21%).

**Figure 3:**
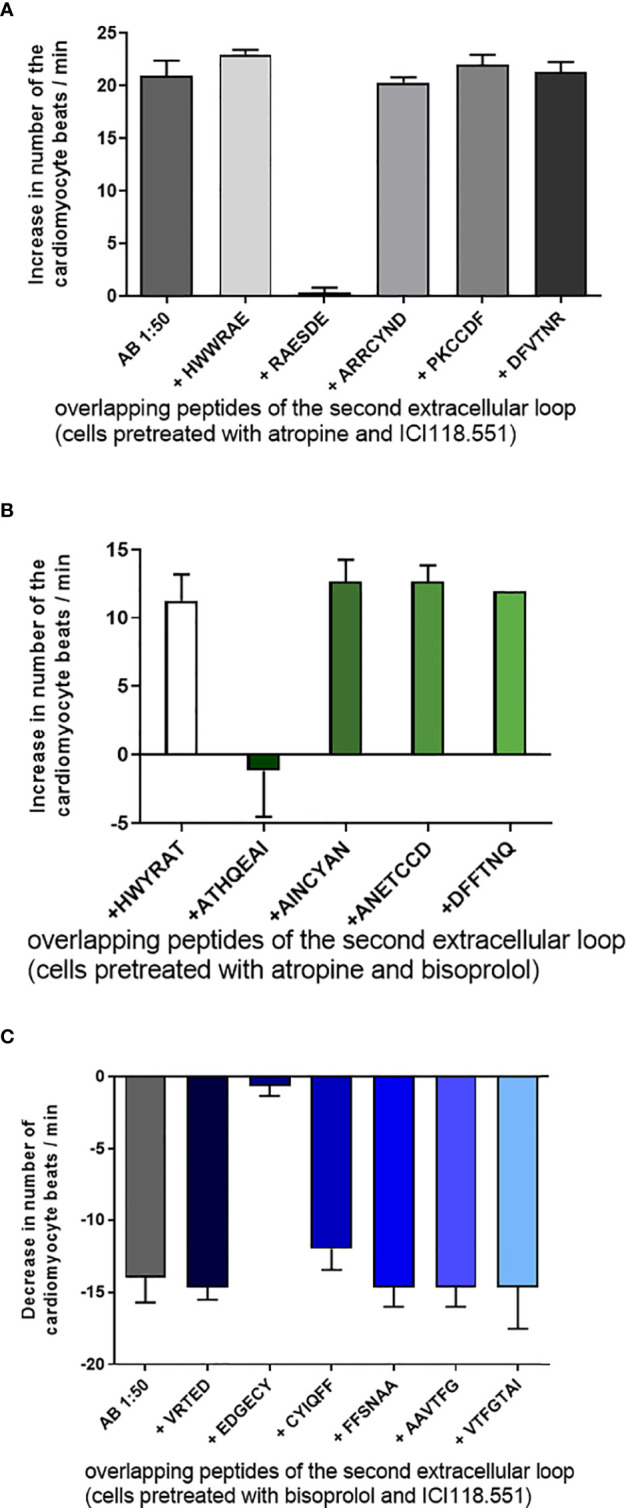
Tagging of the binding epitopes of the antibodies (Abs) on the extracellular structures of the receptors. **(A)** The binding site of the β1-adrenoceptor Abs on the second extracellular loop of receptor represented by the peptide RAESDE. The β2-adrenoceptor Abs recognize the peptide sequence ATHQEAI on the second extracellular loop **(B)** and the muscarinic M2 receptor Abs recognize the sequence EDGECY localized on the second extracellular loop of the M2 receptor **(C)**. The other short overlapping peptides of the three receptors did not develop any neutralizing activity. Cells were pre-treated with bisoprolol, a specific β1- receptor antagonist, ICI118551, a specific β2- receptor antagonist and atropine, a muscarinic M2 receptor antagonist.

To identify the binding site and the epitope of the agonist-like Abs, we pretreated the Abs with short overlapping peptides corresponding to the first or second extracellular loop of the human receptors. **Figure 3A** exhibited the effects of the overlapping peptides corresponding to the second extracellular loop of the β1-AR. Only the peptide RAESDE but not the other four peptides were able to neutralize the activity of the β1-AR Ab. Similar data were observed for the β2-AR (**Figure 3B**) and M2R (**Figure 3C**). The β2-AR Abs were only neutralized by a peptide with the amino acid sequence ATHQEAI (**Figure 2B**). **Figure 2C** shows the identification of binding epitopes of the M2R Ab. The peptide EDGECY localized near the N-terminal part of the second extracellular loop of the M2R abolished the Ab activity completely. Based on these observations, we assume that the three different peptides represent the receptor binding sites of the three different Abs.

## Discussion

Similar to humans, dogs also develop cardiac diseases like CCC (23, 28). In humans, DCM is associated with the formation of autoantibodies against the β1-AR and in some cases, against the M2R (16, 17, 25). In patients with CCC, a correlation is observed among Abs against the β1-AR, β2-AR, and M2R (13, 29). This Abs correlation was also observed in the dogs infected with both Y and Berenice-78 strains of *T. cruzi*. The Abs recognize an epitope on the second extracellular loop of the receptors, which was similar to that observed in patients with the human Chagas disease. We demonstrated that the dogs were able to generate agonist-like Abs against the β1-AR, β2-AR, and M2R. These Abs developed functional properties in a “bioassay” using cultured spontaneously beating neonatal rat cardiomyocytes. These results have also been demonstrated by Daliry et al., who described the formation of the autoantibodies against β1 and M2 receptors in a similar canine model through ELISA method, using VL10, AAS, and Y-strains of *T. cruzi* (28).

In our experiments, we observed that animals infected with the Y-strain developed a higher Ab activity than those infected with the Berenice-78 strain. In particular, the autoantibody activity of the β2-AR and M2R were significantly reduced in Berenice-78 strain-infected animals. These findings underlay the hypothesis that the Y-strain is more aggressive than the Berenice-78 strain (30, 31). The activity of the three identified functional Abs was completely abolished by specific antagonists and peptides corresponding to the second extracellular loops. The binding sites of the Ab on the extracellular loops of the receptor could be identified by using short overlapping peptides corresponding to the second extracellular loop of the three different receptors. The peptide that recognized and neutralized the autoantibody activity represented the binding site on this loop, while other short peptides had no effect on this activity. The epitopes of the autoantibodies against the β1-AR, β2-AR, and M2R in *T. cruzi*-infected dogs are localized near the N-terminal part of the second extracellular loop of the three receptors similar to those observed in human CCC. However, the epitope of the β1-AR autoantibodies found in patients with DCM is different in comparison to that found in patients with CCC. In patients with DCM, the binding site of the β1-AR autoantibodies is localized in the middle cysteine-rich part of the second extracellular loop peptide and represented by the overlapping peptides AINCYAN and ANETCCD. Furthermore, some Abs in patients with DCM recognize their binding site in the first extracellular loop (27). The binding of Abs to the first extracellular loop was never observed in patients and dogs with DCM. The Abs against the β-AR in DCM dogs may play a similar important role as in the DCM Doberman dogs and patients with DCM (32, 33). In patients with DCM, Abs play a very important role in the pathogenesis of this disease. The Abs are removed by immunoadsorption leads in these patients, followed by a long-lasting disappearance of the Abs, an improvement of the cardiac function, and a distinct prolongation of the 5-year survival rate in comparison to the patients with untreated DCM (33, 34). From this data, we assume that the autoantibodies found in *T. cruzi-*infected Beagle dogs may also play an important role in the pathogenesis of cardiac disease in these dogs similar to the role of β1-AR Abs in the pathogenesis of the DCM Doberman dogs.

Autoimmunity in *T. cruzi* infection has played a central role in exploring parasite/host interactions and in the clinical consequences of homologous-overlap Ab production, driven by protozoan antigens against host muscle proteins. The genetic background of *T. cruzi*, along with higher parasitic loads in cardiac and digestive tissues, have been associated with higher production of autoreactive Abs against mammalian proteins, possibly supported by molecular mimicry (12). Clinical and preclinical studies have demonstrated that during cell transplantation, controlling the self-immune response improves cardiac autonomic functionality and promotes a reduction in myocardial inflammation (34–36). In conclusion, our data indicate that the activities of autoantibodies against β-adrenergic and muscarinic receptors might be coordinated by the genetic background of *T. cruzi*, of which higher activities were related to a virulent population of this parasite, with more profound cardiac clinical disturbances. These data warrant further studies on pharmacological and clinical interventions in experimental and human *T. cruzi*-induced cardiomyopathy.

## Data availability statement

The raw data supporting the conclusions of this article will be made available by the authors, without undue reservation.

## Ethics statement

Only freezed sera was used in this experiment, all procedures and experimental protocols were conducted in accordance with the procedures issued by the Brazilian College of Animal Experimentation (COBEA) and approved by the Ethics Committee in Animal Research at UFOP.

## Author contributions

Conceptualization and supervision: GW, AT. Writing-original draft preparation: GW, FB, MOCR, AT. Data curation and resource: FB, JM, VS, GW, MOCR, AT. All authors contributed to the article and approved the submitted version.

## Acknowledgments

Authors thank Dr. MT Bahia (UFOP) that kindly provided the sera for this study. MOCR and AT (Process#305634/2017-8) are in credit to the Conselho Nacional de Desenvolvimento Científico e Tecnológico (CNPq) for the scholarship applied to the development of the research. We also are grateful with UFMG, UFOP, FAPEMIG (APQ-0236-17), CNPq (Process#405946/2021-0) and CAPES for financial support and research scholarship.

## Conflict of interest

Authors GW and JM were employed by company Berlin Cures GmbH.

The remaining authors declare that the research was conducted in the absence of any commercial or financial relationships that could be construed as a potential conflict of interest.

## Publisher’s note

All claims expressed in this article are solely those of the authors and do not necessarily represent those of their affiliated organizations, or those of the publisher, the editors and the reviewers. Any product that may be evaluated in this article, or claim that may be made by its manufacturer, is not guaranteed or endorsed by the publisher.
